# 

**Published:** 1822-08

**Authors:** 


THE LONDON

### Medical and Physical Journal.

2 OF VOL. XLVIII.] AUGUST, 1822. [No. 282.

For many fortunate discoveries in medicine, and for the detection of numerous errors, the world is indebted to the rapid circulation of Monthly Journals; and there never existed any work, to which the Faculty, in Europe and America, were under deeper obligations, than to the Medical and Physical Journal of London, now forming a long, but an invaluable, series.—RUSH.

---

### ADDRESS.

*The Editors beg leave to state, that a considerable part of the present Number was prepared before they entered upon their duties; in consequence of which some articles have been inserted, which, under other circumstances, might have been withheld. They are not sorry, however, that an opportunity is thus afforded of expressing their determination that no personal feelings nor private controversies shall find a place in the London Medical and Physical Journal while it remains under their direction.*

*The Editors have much satisfaction in stating, that they have received the strongest assurances of assistance and support from many of their professional friends; encouragement, by which alone they have been induced to undertake so important and laborious a charge. A reference to the Notice to Correspondents at the end of the Number, will show that several Gentlemen, of the first respectability, have already stepped forward. To them the Editors feel particularly indebted for this early proof of their intention to support the character of a Journal which has enjoyed very extensive patronage for upwards of twenty-four years: at the same time, they beg leave most respectfully, but most earnestly, to recommend the example to others. The superiority of a Journal is not more dependent on the individual exertions of the Editors themselves, than on the assistance afforded by the profession at large; and the present Editors must observe, that, although prepared to make every exertion, they feel, at the same time, that their success must, in a great measure, depend upon others furnishing them with Communications and Intelligence of a nature to interest the medical world.*

The following medical men have died lately:—

Professor Osiander, of Gottingen.
Dr. Reid, author of a work on Nervous Diseases.
Dr. St. Clare, of Preston.

### MONTHLY CATALOGUE OF MEDICAL BOOKS.

Researches respecting the Medical Powers of Chlorine, particularly in Diseases of the Liver; with an Account of a new Mode of applying this Agent, by which its Influence on the System can be secured. By Wm. Wallace, M.R.I.A. Member of the Royal College of Surgeons in Ireland, &c. 8vo. 6s.

Analytic Physiology. By Samuel Hood, M.D. A.B. 8vo. 10s. 6d.

Medico-Chirurgical Transactions. Vol. XII. Part I. With Plates. 9s.

The Dublin Hospital Reports and Communications in Medicine and Surgery. Vol. III. 8vo. 13s. boards.

Gower's (Charles) Description of a Sweating Bath, termed a Sudatorium. 1s.

A Synopsis of the Chemical Decompositions that take place in the Preparations of the London Pharmacopœia. 9d.

### NOTICES TO CORRESPONDENTS.

*We have to acknowledge the receipt of Communications from* Mr. SHAW, Dr. GREGORY, Mr. BROUGHTON, Mr. BAMPFIELD, Mr. SWALLOW *(Staff Surgeon)*, Mr. C. HUTCHISON, Dr. WEBSTER, Mr. HAWARDEN, *of Wigan*, Dr. HANNAY, *of Liverpool*, Dr. ASHBURNER, *and* Dr. JAMES FORBES *(Deputy Inspector of Military Hospitals).*

*We regret that we cannot give insertion to* Mr. L—'s *Communication: the case does not present features of sufficient novelty or practical importance.*

*Our able predecessor in office will perceive that we have taken advantage of the letter from Berlin, with the perusal of which he was so polite as to favour us.*

*We trust that the author of one of the Communications in the present Number will see the propriety of some slight alterations we have made in his Paper.*

*The following Books are on hand for review:*—THOMSON's History of Small-Pox; WOOD's Analytic Physiology; WALLACE on Diseases of the Liver; SWAN on the Nervous System; YEATS on Tic Douloureux; Medecine Pratique, par J. CRUVEILHIER; and Charahteristik der Französischen Medicin mit vergleirhenden Hinblisken auf die Englische, von JOHANN. LUDW. CASPER, M.D. &c. &c. &c.

---

### TO THE READERS OF THE LONDON MEDICAL AND PHYSICAL JOURNAL.

Dr. GRANVILLE *cannot withdraw from the office of Editor of the Medical and Physical Journal, without addressing a few observations to his readers.*

*Having undertaken the conduct of this valuable Miscellany at the suggestion of his learned predecessor, Dr. Granville (though scarcely able to spare sufficient time for such a charge, accepted the office,) with the faint prospect of the Journal being again, at some period not far distant, replaced into the same able hands. That prospect, however, existing no longer, in consequence of the final departure of* Dr. HUTCHINSON *from Paris to Russia; and Dr. Granville's present professional occupations rendering it absolutely impossible for him to continue in its office, he retires from it, from a conviction that, in arranging a publication of such magnitude, without assistance, the Editor should be wholly free from every other engagement.*

*The repeated, and often unexpected, calls on the time of one engaged in the arduous duties of private and public practice, seem incompatible with the many serious objects which must necessarily occupy the attention of the Editor of a popular Journal. This explanation is not intruded wantonly on the public; but is given with a view to obviate any charge of fickleness.*

*During the last six months, more especially, Dr. Granville has studied to render the London Medical and Physical Journal still more worthy of that patronage which the public have never failed to grant to it. He has given method to the arrangement of its contents,—added several new departments of medical and scientific information,—collected valuable original papers,—varied the articles of compilation,—given extensive, as well as more numerous, Reviews of both English and Foreign Works,—and, lastly, brought together such a vast number of important and various subjects of Intelligence as no other contemporary Journal has ever collected together in any of its Numbers.*

*In effecting all this, Dr. Granville has to boast of nothing but industry; and he trusts that, in consideration of the desire he has manifested of doing good, his readers will forgive him the want of talent, and of every other qualification necessary to effect it.* —Valete.

*Saville-row; July,* 1822.

